# Reduced hatchability of *Anopheles gambiae* s.s eggs in presence of third instar larvae

**DOI:** 10.1186/1756-0500-7-231

**Published:** 2014-04-11

**Authors:** Gilbert M Gotifrid, Felister M Urasa, Gerald Katunzi, Jacob G Yarro, Stephen Munga, Eliningaya J Kweka

**Affiliations:** 1 Department of Zoology and Wildlife Conservation, University of Dar-es-salaam, College of natural and applied sciences, Dar es Salaam, Tanzania; 2 Kenya Medical Research Institute, Centre for Global Health Research, P.O.Box 1578, Kisumu, Kenya; 3 Division of Livestock and Human Diseases Vector Control, Mosquito Section, Tropical Pesticides Research Institute, P.O.Box 3024, Arusha, Tanzania; 4 Department of Medical Parasitology and Entomology, Catholic University of Health and Allied Sciences, P.O. Box 1464, Mwanza, Tanzania

**Keywords:** *Anopheles gambiae* s.s, Microcosms, Hatchability, Larvae

## Abstract

**Background:**

We investigated the hatchability rates of freshly laid *Anopheles gambiae* s.s. eggs in presence of third instars larvae. These experiments were conducted using 30 eggs in larval densities of 20, 60 and 100 larvae in microcosms. These experiments were designed to evaluate the eggs hatchability in habitats with late larvae instars of the same species (experimental) or no larvae at all (control). Freshly laid eggs of *An.gambiae* s.s. were washed in microcosms containing larvae of third instars in different three densities (20, 60 and 100) and likewise in control microcosms (without larvae). Eggs hatchability was monitored twice daily until no more first instar larvae emerged. The numbers of first instars larvae were recorded daily and lost eggs were considered preyed upon by third instars.

**Findings:**

The findings of this study showed that egg hatchability was significantly influenced by larval density.

**Conclusion:**

The findings of this study suggest that presence of larvae in habitats may significantly reduce hatchability of eggs.

## Findings

**Figure 1:**
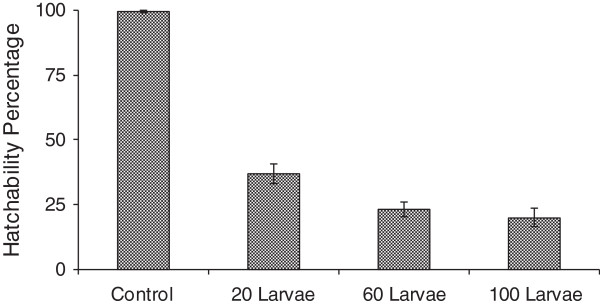
**Hatchability response of *Anopheles gambiae *** **s.s eggs in control and different larvae densities.**

Eggs hatching were observed to take place on the second day after being washed in the microcosms. Eggs hatchability in control experiments was 99.4% and in experimental microcosms hatchability varied with larvae density, in larval density of 20 (37.2%); in larval density of 60 (23.2%) and in 100 larval density (20.2%). The eggs hatchability in different larval densities and control is shown in Figure 1. Egg hatchability in the three densities of larvae used had statistically significantly different (F = 641.67, DF = 2, *P* < 0.001). When compared using Tukey HSD post hoc tests; egg hatchability in density of 20 larvae per oviposition substrate was significantly different to control (*P* < 0.001); likewise for densities of 60 larvae (P < 0.001) and 100 larvae (*P* < 0.001) (Table 1 and Figure 1). The findings of this study have demonstrated that *An.gambiae* eggs hatchability reduction are influenced by many factors including predation by late instars and predators, available microbial community and habitats types [1]. Eggs hatchability in this study was larvae density dependent. In non-parental care animals the offspring’s survival and development is dependent mostly on the quality of the habitat where the eggs are laid [2-4]. Gravid female of *Anopheles gambiae* s.s. (hereafter referred to as *An.gambiae*) mosquitoes have been reported to have species specific oviposition site choices [5-7]. A previous study showed that egg hatchability in *An.gambiae* eggs was influenced by several factors such as temperature , salinity of water and humidity [8]. Organic substances of environmental origin and bacteria have been also suggested to influence oviposition site selection and eggs hatchability for different species [9,10]. Similarly, other studies demonstrated that, aged water or water with crowded larvae received significantly fewer *Aedes aegypti* and *An. gambiae* eggs than control water which had no larvae [11-13]. In one study in western Kenya, *An. gambiae* species laid significantly more eggs in habitats without larvae than habitats with larvae [14]. The main plausible reason in such situations is to avoid predation on eggs or first larval instars by late instars [15,16] or food resources availability/scarcity and competition [15,17]. In other experiments, it has been demonstrated that the existence of different larval instar in the same habitat, the early instars are predated by late instars [16]. The occurrence of the same instars of the different species have been demonstrated to have competition for resources but no predation was observed [16,18]. Currently, there is limited information on what would happen if the gravid mosquitoes lay eggs in habitats with late instar larvae. Little is known about what happens to eggs after being laid in habitats with late instars of the same species. In our previous studies in western Kenya involving choice experiments, *An. gambiae* was found to prefer laying significantly more eggs in habitats without mosquito larvae [13]. Therefore, this study assessed the hatchability of freshly laid eggs in microcosms with different densities of third instar larvae of *An. gambiae* s.s. Insects have chemoreceptor's which are useful for detection of predators risk in habitats [19].The hatchability of eggs laid in natural population of mosquito habitats have seen to be at risk of late instars for having the microbial layer on shells which could be food source for the late instars [20,21]. Those bacteria play a major role in the eggs shell breaking during hatching process and adult oviposition mediation chemical cues productions [1]. It is hypothesized that, the observed density dependent egg hatchability was caused by the presence of these bacteria which may have attracted the predation of eggs. Delay or reduction in egg hatchability may affect individual fitness, population structure and dynamics [22]. Larval density dependent egg hatchability results in the current study further suggests why mosquitoes might skip habitats with late instars larvae. Additionally, this could imply ability to detect resource limitations in such habitats by gravid mosquitoes.

**Table 1:** Hatchability comparison of eggs between controls bowls and bowls with different larvae densities

**Larvae density**	**Number of eggs**	**Percentage egg hatchability**	**% hatchability reduction**
0	30	99.4	0.0^a^
20	30	37.2	62.2^b^
60	30	23.2	76.2^c^
100	30	20.2	79.2^c^

NOTE: In percentage hatchability reduction, the numbers with different superscript letters in the same column, they differ statistically significant.

## Methods

Study was conducted at Tropical Pesticides Research Institute Insectary, based in Arusha Tanzania for two months.

### Adult mosquitoes rearing and eggs laying

Three days old females of *An. gambiae* s.s post emergence mosquitoes were fed on rabbit for 30 minutes. Blood fed females were then kept in insectary at a temperature of 27 ± 2°C, Relative humidity 78 ± 2% and light 12 L: 12D. The gravid females after 72 hrs post feeding were given a wet filter paper in a cage to act as oviposition substrate. The eggs laid were used immediately for these experiments.

### Hatchability experimental set up

Experiments were set up in white microcosms having a diameter of 16.7 cm and depth of 1.7 cm. The sides of the microcosms just at the level of the water were lined with white paper to prevent the eggs from adhering to the surface of the microcosm and drying up. Freshly laid eggs on filter papers were washed in microcosms with dechlorinated water with third instar larvae in three densities of 20, 60 and 100. In the control arm, eggs were washed in microcosms without larvae and in both experiments; hatchability was monitored for three days. Hatched first instar larvae were collected and taken out of the microcosms every two hours’ time. Insectary temperature was maintained at 27 ± 2°C and relative humidity was 78 ± 2%. Thirty freshly laid eggs of *An. gambiae* s.s. were introduced in each microcosm in all three densities of *An.gambiae* s.s larvae. Each experiment had six replicates for each density and control.

### Data analysis

Data were analyzed using SPSS 17.0 (SPSS Inc., Chicago, IL). Comparison of the mean number of hatched eggs was compared by ANOVA between the larvae densities in treatments and control. The significance level for the means of the three densities of 20, 60 and 100 were separated by Tukey HSD test.

### Ethical approval

The study was approved by Tropical Pesticides Research Institute (TPRI), Proposal review and ethical committee. The use of rabbit for feeding mosquitoes was approved as a daily routine permission in mosquito colony maintenance at TPRI.

## Conclusion

This study has shown that the existence of the third instar larvae in breeding sites affect egg hatchability but also survivorship of the newly hatched first instars. More studies have to be done in semi field environment to determine egg hatchability in more complex environments and investigation of larva produced chemical factors (cuticle exudates) that play the role of emergence inhibitors of conspecific eggs is on progress.

## Competing interest

Authors declare to have no competing interest. We have no financial or non-financial competing interests.

## Authors’ contributions

EJK and SM conceived the study and did data analysis interpretation and wrote the manuscript. GMG and GK, performed experiments and data handling. EJK, SM, GJY and FU revised the Manuscript. All authors have accepted the submission of this paper. All authors read and approved the final version of this manuscript.
